# Urinary exosomal viral microRNA as a marker of BK virus nephropathy in kidney transplant recipients

**DOI:** 10.1371/journal.pone.0190068

**Published:** 2017-12-21

**Authors:** Myeong Hee Kim, Yu Ho Lee, Jung-Woo Seo, Haena Moon, Jin Sug Kim, Yang Gyun Kim, Kyung-Hwan Jeong, Ju-Young Moon, Tae Won Lee, Chun-Gyoo Ihm, Chan-Duck Kim, Jae Berm Park, Byung Ha Chung, Young-Hoon Kim, Sang-Ho Lee

**Affiliations:** 1 Department of Laboratory Medicine, Kyung Hee University, Seoul, Korea; 2 Division of Nephrology, Department of Internal Medicine, Kyung Hee University, Seoul, Korea; 3 Division of Nephrology, Department of Internal Medicine, Kyungpook National University Hospital, Daegu, Korea; 4 Department of Surgery, Samsung Medical Center, Seoul, Korea; 5 Division of Nephrology, Department of Internal Medicine, College of Medicine, The St. Mary’s Hospital of Catholic University of Korea, Seoul, Korea; 6 Division of Nephrology, Department of Internal Medicine, College of Medicine, Inje University, Pusan, Korea; Gustave Roussy, FRANCE

## Abstract

**Objective:**

Bkv-miR-B1-5p, one of the microRNAs encoded by BK virus, was recently reported to be elevated in the blood among the patients with BK virus nephropathy (BKVN). Urinary exosome was suggested to be a possible source of biomarker for kidney diseases, but it was unknown whether it could contain viral microRNA as well as human microRNAs. The aim of this study was to evaluate whether urinary exosomal BK viral microRNA were expressed during replication and could be used to diagnose BKVN in kidney transplant recipients.

**Materials and methods:**

In a cross-sectional multicenter study, we collected and analyzed 458 graft biopsies from 385 kidney transplant recipients. Urine samples were collected at the time of graft biopsy, and microRNAs in urinary exosome were measured once. For 13 patients with BKVN and 67 age, sex-matched kidney transplant recipients, we measured BK viral microRNA B1-5p, 3p and human microRNA-16 in urinary exosomal fraction and compared the diagnostic value with BK viral load in plasma and urine.

**Results:**

Pathology proven BKVN was diagnosed in 13 patients (2.8%). High levels of bkv-miR-B1-5p and bkv-miR-B1-3p were shown in all patients with BKVN. Meanwhile, plasma BK viral load assay (cut-off value of ≥ 4.0 log_10_ copies/mL) showed false negative in 3 cases and urinary BK viral load assay (cut-off value of ≥ 7.0 log_10_ copies/mL) showed false negative in 1 case among these 13 patients. The receiver operator characteristics curve analysis for bkv-miR-B1-5p and bkv-miR-B1-5p/miR-16 showed excellent discriminative power for the diagnosis of BKVN, with area under the curve values of 0.989 and 0.985, respectively.

**Conclusions:**

This study suggests that urinary exosomal bkv-miR-B1-5p and bkv-miR-B1-5p/miR-16 could be surrogate markers for the diagnosis of BKVN.

## Introduction

BK virus (BKV) is a major causative agent of nephropathy in kidney transplant recipients. BK viral nephropathy (BKVN) can lead to deterioration of the transplanted kidney and graft failure [1]. The prevalence of biopsy proven BKVN is gradually decreasing with the application of active molecular surveillance using BK viral load assay in plasma and urine samples [2]. However, substantial proportion of kidney transplant recipients still experiences graft injury due to clinically significant BKVN, with the infection rate between 1% and 10% and the frequencies of graft loss between 10% and 80% [1].

Recent studies have demonstrated that microRNAs encoded by BKV can also be detected in the blood and urine of patients with BKV infection [3–5]. MicroRNAs are non-coding RNAs of 20–22 nucleotides. The microRNAs regulate gene expression through degradation of messenger RNA or translational inhibition [5]. Members of several virus families have been reported to encode microRNAs [6]. BKV-encoded microRNAs target the viral protein T antigen with perfect complementarity to the 3p coding end of the T antigen messenger RNA [4].

Urinary exosomes are a subset of extracellular vesicles derived from the inward budding of endosomal membranes [7]. Urinary exosomes may concentrate potential biomarkers of kidney diseases to reflect the pathophysiological state of the renal system [8]. However, it is unclear that exosomal BKV microRNAs can be detected in urine samples from infected patients.

In this study, we assessed the prevalence of biopsy proven BKVN in a cross-sectional multicenter study. Further, we evaluated whether urinary exosomal BKV microRNAs were expressed during replication and could be used to diagnose BKVN in kidney transplant recipients.

## Materials and methods

### Study populations

Patients with BKVN were chosen from ARTKT-1 (assessment of immunologic risk and tolerance in kidney transplantation) study, which was a cross-sectional sample collection study for kidney transplant recipients who underwent allograft biopsy at six different centers (Kyung Hee University Hospital at Gangdong, Kyung Hee University Hospital, Kyungpook National University Hospital, Samsung Medical Center, St. Mary’s Hospital of Catholic University of Korea, and Inje university Busan Paik hospital) from August 2013 to July 2015. All biopsied specimens were scored and diagnosed according to the Banff 2007 scoring system [9]. The diagnosis of BKVN was confirmed by renal allograft biopsy; positive simian virus 40 immunohistochemical stains, the presence of viral cytopathic effects in renal tubular cells with interstitial mononuclear inflammatory cell infiltrates and/or the presence of homogenous intranuclear viral inclusions. Pathologic patterns of BKVN were defined based on the previous study [10]; pattern A, cytopathic/cytolytic changes with absent or minimal inflammation; pattern B, cytopathic/cytolytic changes associated with patchy or diffuse tubulointerstitial inflammation and atrophy; and pattern C, graft sclerosis with features of extensive interstitial fibrosis/tubular atrophy and lymphocytic inflammation. We performed active molecular surveillance by measuring urinary or plasma BK viral load every three months during the first year and every six months during the second year after KT, according to previous recommendations [2, 11]. Plasma and urinary BK viral load was also checked when renal allograft dysfunction was found and/or renal allograft biopsy was performed.

Patient’s characteristics and laboratory results are presented in Table 1. Estimated glomerular filtration rate (eGFR) was calculated using the modification of diet in renal disease (MDRD) formula. This study was approved by the local institutional review board (#2012-01-030, Kyung Hee Neo Medical Center [KHNMC]). Written informed consent was obtained from all patients.

**Table 1:** Clinical characteristics of patients with BK virus nephropathy and other kidney transplant recipients.

	BKVN (n = 13)	Others (n = 445)	*p* value
Age (years)	46.7±15.8	46.9±11.7	0.951
Sex (male: female)	9:4	296:149	0.838
Duration after KT (days)	321±314	748±1377	<0.001
Pathologic diagnosis (n, %)		Normal (142, 31.9%) TCMR (76, 17.2%) Suspicious for TCMR (55, 12.4%) ABMR (64, 14.3%) ATN (31, 7.0%) GN (29, 6.5%) CNI toxicity (26, 5.8%) Others* (22, 4.9%)	
Pathologic patterns of BKVN (10) (n, %)	Pattern A (4, 30.8%) Pattern B (6, 46.1%) Not specified (3, 23.1%)		
Creatinine (mg/dL)	2.30±0.92	2.05±1.69	0.599
eGFR (ml/min/1.73m^2^)	30.96±9.00	46.57±24.85	<0.001
Urine PCR (mg/gCr)	407.8±482.7	890.4±1781.7	0.351

Abbreviations: BKVN, BK virus nephropathy; KT, kidney transplantation; eGFR, estimated glomerular filtration rate; PCR, protein-to-creatinine ratio; TCMR, T-cell mediated rejection; ABMR, antibody-mediated rejection; ATN, acute tubular necrosis; GN, glomerulonephritis; CNI, calcineurin inhibitor

*Others include nonspecific interstitial fibrosis and tubular atrophy, hypertensive nephrosclerosis, diabetic nephropathy and acute interstitial nephritis

### Collection of specimens

Mid-stream, second morning void urine samples from kidney transplant patients were collected at the day of graft biopsy. The urine specimens were centrifuged at 2,000g at room temperature for 20 min, and the supernatants and pellets were separated and stored at -80°C until use. Plasma sample was also collected at the same time and stored at -80°C until use.

### RNA isolation from urinary exosomes and quantification of microRNA

Spin column-based isolation of exosomal RNA from 1mL of urine was performed using exoRNeasy Serum/Plasma midi kits (QIAGEN GmbH, Hilden, Germany) according to the manufacturer’s instructions. There was no DNA contamination and the integrity of the RNA was confirmed with agarose gel electrophoresis and Agilent 2100 Bioanalyzer. RNA was stored at -80°C until use. BKV microRNA expression was assessed by quantitative real-time reverse transcriptase PCR (qRT-PCR) using human TaqMan microRNA assays (Applied Biosystems, Foster City, CA). Reactions using 3 μL of RNA were performed with TaqMan microRNA Reverse Transcription Kit and TaqMan microRNA-specific primers (assay IDs: hsa-miR-16:000391, bkv-miRB1-5p:007796, bkv-miR-B1-3p:006801; Applied Biosystems). The qRT-PCR reaction contained 1 μL of reverse transcription product, 1x TaqMan Universal PCR mastermix, no AmpErase UNG, and 1 μL of primer mix. The complementary DNA (cDNA) was amplified using an ABI StepOnePlus real-time PCR system (Applied Biosystems). Reactions were incubated at 95°C for 10 min, followed by 40 cycles at 95°C for 15 s and 60°C for 60 s. A control sample was spiked with known concentrations of synthetic BKV microRNA mimic duplex oligonucleotides (bkv-miR-B1; IDT, Coralville, IA, USA). The sequences of the duplexes were 5’-AUCUGAGACUUGGGAAGAGCAU and 5’-UGCUUGAUCCAUGUCCAGAGUC [5].

### Quantitative real-time polymerase chain reaction (PCR) of BK viral load

DNA extracts from urine and plasma were prepared using the AccuPrep^®^ Viral DNA Extraction Kit (Bioneer Co., Gyeonggi, Korea). For the AccuPower^®^ BKV Quantitative PCR assay (Bioneer Co.), 5 μL of DNA extract was mixed with 45 μL of master mix, and real-time PCR was performed using a PCR thermocycler (Exicycler 96 Real-Time Quantitative Thermal Block; Bioneer Co.), under the following conditions: pre-denaturation at 95°C for 5 min, 45 cycles of denaturation at 95°C for 5s, and annealing/extension at 55°C for 5s. An internal positive control consisting of DNA sequences unrelated to the BKV target sequence was co-amplified in each reaction to determine whether PCR was inhibited by the sample. All procedures were performed according to the manufacturer's instructions.

### Statistical analysis

To compare the patient characteristics and BKV microRNA expression across all five clinical groups, we used one-way analysis of variance and Pearson’s χ^2^ test. The levels of urinary exosomal BKV microRNAs were expressed and analyzed after log_10_ transformation since these data was non-normally distributed. Pearson correlation analysis was used to compare the levels of BKV microRNA and BK viral load. Receiver operating characteristic (ROC) curves were generated, and areas under the curve (AUC) were calculated to assess the diagnostic power of BKV microRNA and BK viral load titers to distinguish patients with BKVN. The optimal cut-off point of each measure was determined by Youden's J statistic in order to provide both high sensitivity and high specificity. Data are expressed as the mean ± standard deviation (SD) or as the number of patients and percentages. *p* values under 0.05 were considered statistically significant.

## Results

### The prevalence of BKVN in kidney transplant recipients

The diagnosis of BKVN was made in 13 patients by pathologic confirmation (Table 1). The prevalence was 2.8% of all investigated allograft biopsies (13/458) and 4.8% of indication biopsies (12/249). Only two centers performed protocol biopsy for the patients who agreed with the consent form (at 3 week and 6 month after kidney transplantation in Kyung Hee University at Gandong, at 3 month after kidney transplantation in St. Mary’s Hospital of Catholic University, respectively), and the prevalence of BKVN among patients who underwent protocol biopsy was 0.5% (1/209). The mean age of patients with BKVN was 46.7 years, the mean time for the diagnosis was 321±314 days after kidney transplantation and the mean eGFR at the time of biopsy was 36.09±9.00 ml/min/1.73m^2^.

### Comparison of the levels of viral microRNAs between urinary supernatant and exosomal fraction

**Fig 1:**
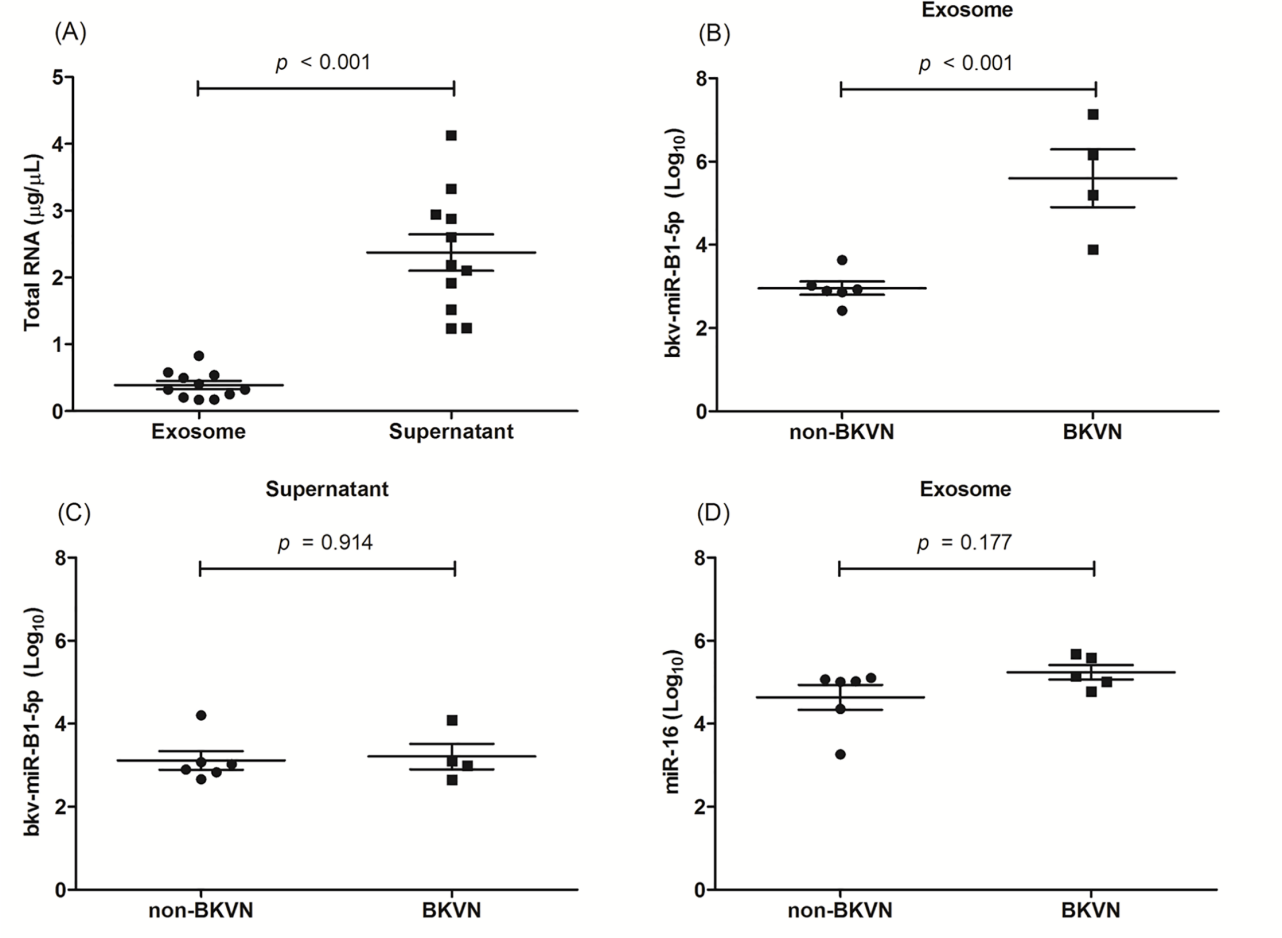
Comparison of the levels of viral microRNAs between urinary supernatant and exosomal fraction. **(A)** The levels of total RNA in urinary exosomal fraction and urinary supernatant. **(B) and (C)** The levels of bkv-miR-5p in patients without BKVN and those with BKVN. (B) indicates the levels of bkv-miR-5p in urinary exosomal fraction, and (C) indicates those in urinary supernatant. **(D)** The levels of miR-16 in urinary exosomal fraction of patients with BKVN and those without BKVN.

To determine the distributions of viral microRNAs in urine, we first measured the levels of total RNA, bkv-miR-B1-5p and miR-16 in urinary supernatant and exosomal enrichment fraction, respectively, in 5 patients with BKVN and 6 patients without any evidence of BK viral replication. The concentration of total RNA was significantly lower in urinary exosomal enrichment fraction than that in urinary supernatant (Fig 1A). However, the levels of bkv-miR-B1-5p in urinary exosomal fraction were significantly higher in patients with BKVN, while those levels in urinary supernatant were not different between two groups (Fig 1B and 1C). Furthermore, miR-16, a known surrogate marker for vesicular microRNAs [12], was detectable in urinary exosomal fraction of all patients and the levels of miR-16 were not different between the patients with BKVN and those without BKVN (Fig 1D). In contrary, miR-16 in urine supernatant was detected only in 36.4% of patients (4/11). Taken together, this preliminary data suggested that urinary exosomal fraction, rather than urinary supernatant, would be appropriate for the assessment of BKV microRNAs.

### Comparison of urinary exosomal BKV microRNAs with plasma and urinary BK viral load

To compare viral microRNA in exosomal fraction and BK viral load in plasma and urine with those in patients with other pathology, 67 age and sex-matched renal transplant recipients were selected regardless of BK viremia or viruria among patients with Banff classification of 1, 2 and 4 on graft biopsy. The clinical and laboratory characteristics of enrolled patients are shown in Table 2. Percentage of ABO incompatible kidney transplantation, number of HLA mismatches and the use of immunosuppressive agents were not different across the groups, but the patients with BKVN and transplant rejection (both acute and chronic) showed lower eGFR than in patients with normal pathology.

**Table 2:** Clinical characteristics, BK viral load and urinary exosomal BK virus microRNA in 13 patients with BKVN and 67 age, sex-matched kidney transplant recipients.

	BKVN	Normal	TCMR	Acute ABMR	Chronic active ABMR	p value
Number of patients	13	15	27	9	16	
Age (years)	46.7±15.8	51.7±10.9	47.6±10.7	47.0±8.0	49.5±9.2	0.727
Sex (male: female)	9:4	10:5	18:9	7:2	11:5	0.980
Duration after KT (days)	321±314	138±46	479±662	1129±1038	3257±1895	<0.001
ABO incompatible KT (n, %)	2 (15.4)	1 (6.7)	4 (14.8)	2 (22.2)	0 (0)	0.862
HLA mismatching (n)	3.6±1.6	4.5±1.3	3.5±1.6	3.4±1.7	3.1±1.5	0.638
Induction immunosuppression						
Basiliximab	10 (76.9)	14 (93.3)	20 (74.1)	8 (88.9)	14 (87.5)	0.750
Anti-thymocyte globulin	3 (23.1)	1 (6.7)	7 (25.9)	1 (11.1)	2 (12.5)	
Maintenance immunosuppression						
Steroid (n, %)	12 (92.3)	15 (100)	25 (92.6)	9 (100)	15 (93.8)	0.764
Tacrolimus (n, %)	12 (92.3)	14 (93.3)	21 (77.8)	6 (66.7)	12 (75.0)	0.359
Cyclosporine (n, %)	0 (0)	0 (0)	5 (18.5)	2 (22.2)	3 (18.8)	0.182
Mycophenolate mofetil (n, %)	9 (69.2)	12 (80.0)	18 (66.7)	8 (88.9)	9 (69.2)	0.696
mTOR inhibitor (n, %)	1 (7.7)	1 (6.7)	0 (0)	1 (11.1)	0 (0)	0.782
Serum creatinine (mg/dL)	2.30±0.92	0.85±0.10	2.88±2.57	3.08±1.50	2.88±1.59	<0.001
eGFR (mL/min/1.73 m^2^)	23.3±3.0	77.5±18.3	33.7±17.2	26.6±13.4	27.2±13.0	<0.001
Plasma BK viral load (copies/mL)*	4.27±1.45	1.26±0.0	1.26±0.0	1.89±1.30	1.33±0.30	<0.001
Urinary BK viral load (copies/mL)*	7.73±2.06	1.63±0.65	2.21±1.60	3.42±3.07	2.27±1.55	<0.001
Exosomal bkv-miR-B1-5p*	7.01±0.66	2.55±0.83	2.62±0.88	2.95±2.08	2.36±0.77	<0.001
Exosomal bkv-miR-B1-3p*	8.05±0.93	3.88±1.38	3.18±1.34	3.76±2.15	3.33±1.28	<0.001
Exosomal miR-16*	5.25±0.39	4.81±0.40	4.85±0.48	4.79±0.33	4.92±0.42	0.080

Abbreviations: BKVN, BK virus nephropathy; TCMR, T-cell mediated rejection; ABMR, acute antibody-mediated rejection; KT, kidney transplantation; HLA, human leukocyte antigen; mTOR, mechanistic target of rapamycin; eGFR, estimated glomerular filtration rate.

Values are presented as mean ± standard deviation or number of patients with percentages.

*The levels of BK viral load and microRNAs were calculated and expressed after log_10_ transformation.

**Fig 2:**
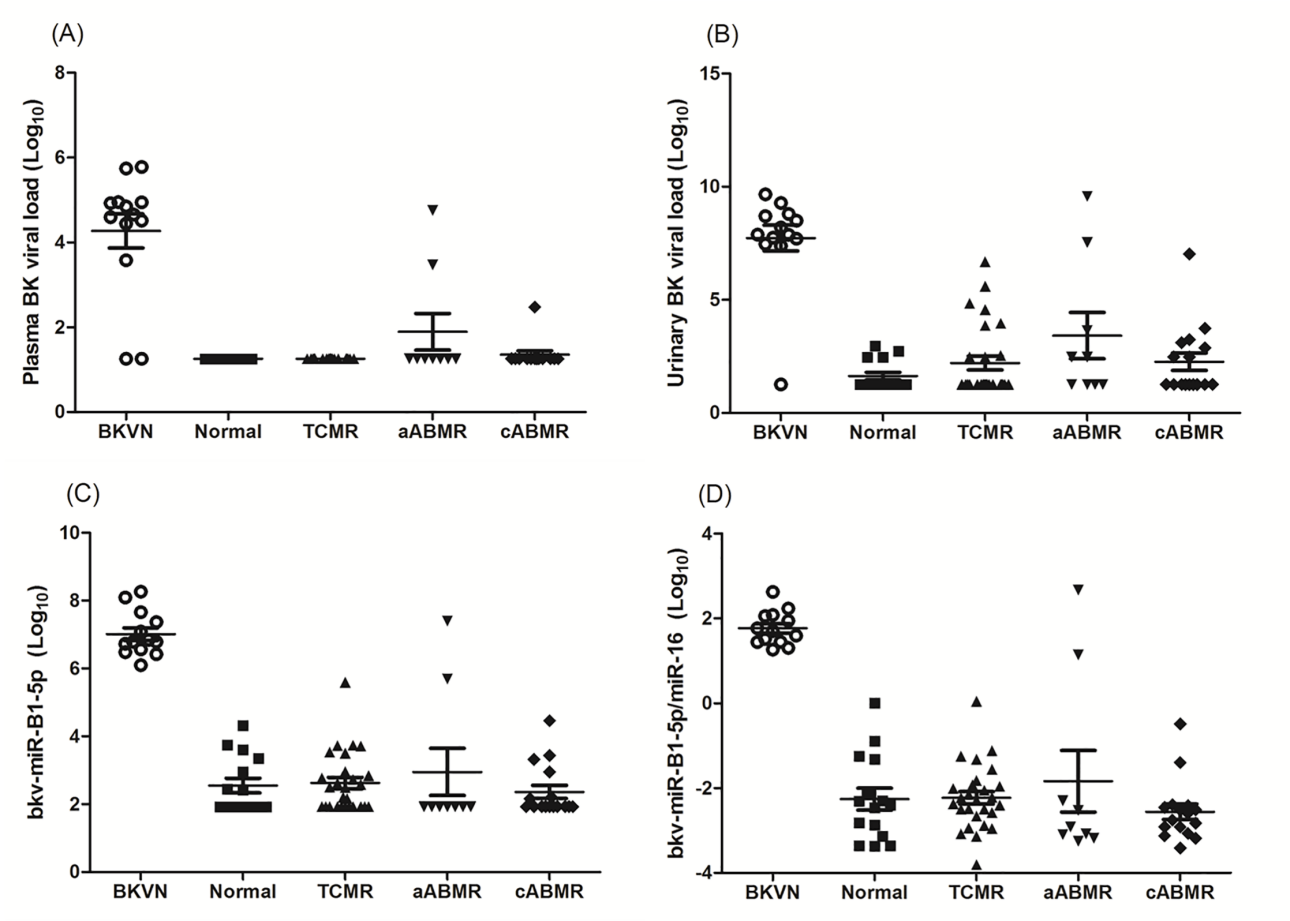
Plasma BK viral load, urinary BK viral load, urinary exosomal bkv-miR-B1-5p, and bkv-miR-B1-5p/miR-16 levels according to renal allograft status. **(A) and (B)** Plasma and urinary BK viral load was elevated in patients with BKVN. However, two patients with BKVN showed negative plasma BK viral load, and one patient showed negative urinary BK viral load **(C) and (D)** Patients with BKVN also showed highest levels of bkv-miR-B1-5p and bkv-miR-B1-5p/miR-16 (*p*<0.001). All patients in this group revealed positive urinary BK viral microRNA levels. Abbreviations; TCMR, T-cell mediated rejection; aABMR, acute antibody-mediated rejection; cABMR, chronic active antibody-mediated rejection; BKVN, BK virus nephropathy.

Both urinary exosomal bkv-miR-5p and bkv-miR-B1-3p levels were significantly increased in patients with BKVN (Table 2 and Fig 2). On the other hand, the levels of miR-16 were not significantly different between the patients with BKVN and those with normal pathology. The bkv-miR-B1-5p was a specific microRNA used to distinguish BKV from JC virus (JCV) [5]. Therefore, we focused on bkv-miR-B1-5p PCR assay. Plasma BK viral load, urinary BK viral load, and the levels of bkv-miR-B1-5p and bkv-miR-B1-5p/miR-16 are shown in Fig 2, according to different groups.

**Fig 3:**
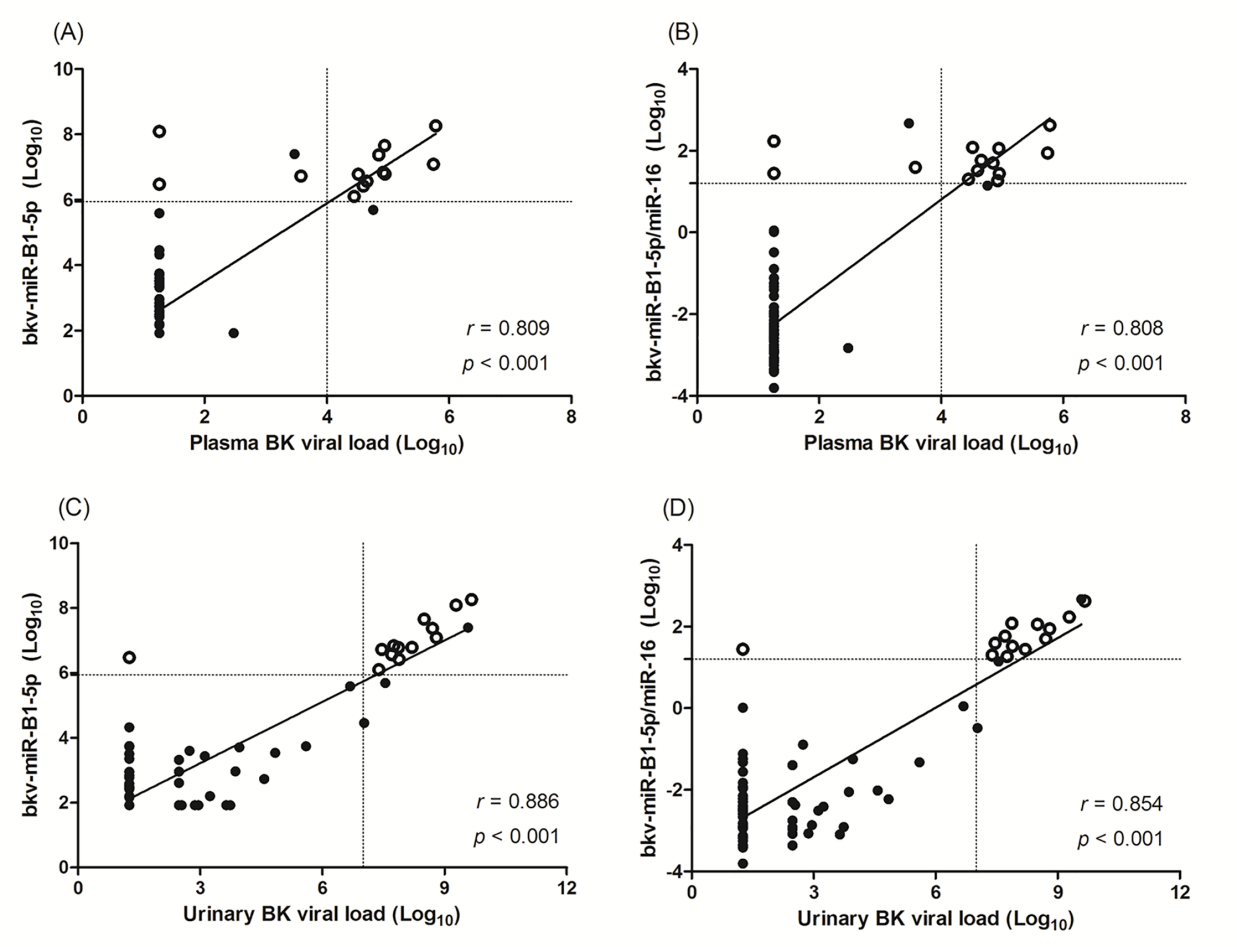
Correlation analyses between urinary exosomal bkv-miR-B1-5p, bkv-miR-B1-5p/miR-16 and BK viral load. **(A) and (B)** A significant positive correlation between bkv-miR-B1-5p and plasma BK viral load was shown. **(C) and (D)** There was also a positive correlation between bkv-miR-B1-5p and urinary BK viral load. Open circles represent patients with BKVN and closed circles represent the remaining patients. Dotted lines signify the cut-off values for each test in this study (5.9 log_10_ copies/mL for bkv-miR-B1-5p, 1.2 log_10_ copies/mL for bkv-miR-B1-5p/miR-16, 4.0 log_10_ copies/mL for plasma BK viral load, and 7.0 log_10_ copies/mL for urinary BK viral load).

Although BK viral load was also detected at a high level in most patients with BKVN, currently recommended cut-off value of plasma BK viral load (≥4.0 log_10_ copies/mL) underestimated the diagnosis of BKVN (three false negative cases with plasma BK viral load) (Fig 3) [13]. Diagnosis of BKVN based on urinary BK viral load (≥7.0 log_10_ copies/mL) also showed false negative in 1 case among 13 patients with BKVN. Among 67 patients without BKVN, false positive was only one case in plasma DNA assay, but three cases were found to be false positive with urinary DNA assay.

The levels of bkv-miR-B1-5p and bkv-miR-B1-5p/miR-16 showed a significant positive correlation with urinary BK viral load (r = 0.886 and 0.854, respectively) (Fig 3). A slightly lesser, but significant degree of correlation was also found between bkv-miR-B1-5p, bkv-miR-B1-5p/miR-16 and plasma BK viral load (r = 0.809 and 0.808, respectively).

### ROC curve analysis to evaluate the ability of urinary exosomal microRNA levels to distinguish patients with BKVN

**Fig 4:**
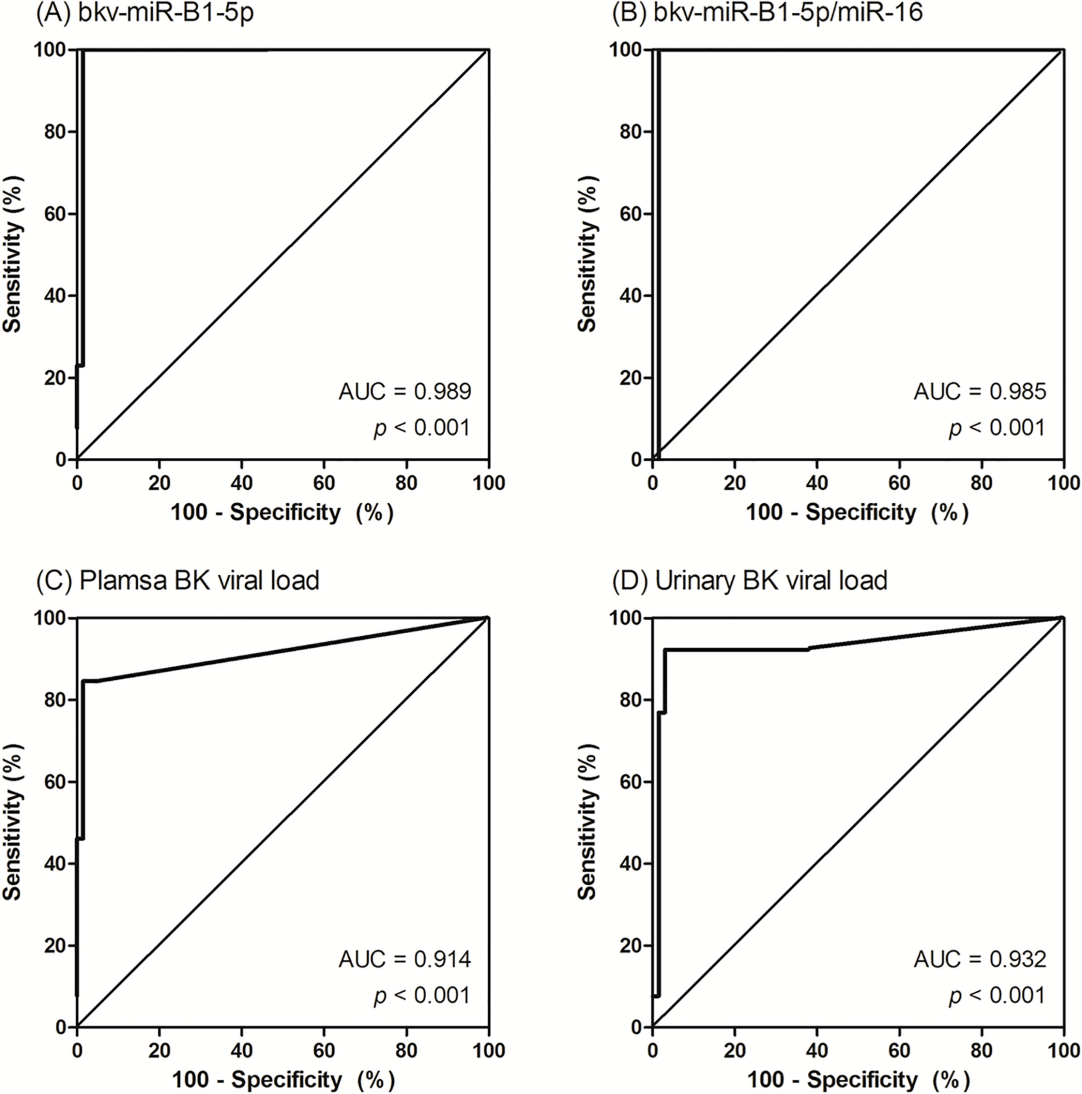
**Receiver operating characteristic (ROC) curves for the diagnosis of BK virus nephropathy (BKVN) using (A) bkv-miR-B1-5p, (B) bkv-miR-B1-5p/miR-16, (C) plasma BK viral load, and (D) urinary BK viral load.** ROC curves demonstrating diagnostic accuracy for detecting patients with BKVN by area under the curve (AUC). The AUC for bkv-miR-B1-5p, bkv-miR-B1-5p/miR-16, plasma BK viral load, and urinary BK viral load are 0.989, 0.985, 0.914, and 0.932, respectively.

The AUC from ROC curve analysis using data from the 80 subjects showed excellent results; the AUC was 0.989 for bkv-miR-B1-5p (95% confidence interval [CI] 0.934–1.000), 0.985 for bkv-miR-B1-5p/miR-16 (95% CI 0.928–0.999), 0.914 for plasma BK viral load (95% CI 0.830–0.965) and 0.932 for urinary BK viral load (95% CI 0.853–0.976) (Fig 4). The cut-off values for bkv-miR-B1-5p and bkv-miR-B1-5p/miR-16 were 5.9 log_10_ copies/mL (sensitivity, 100%; specificity, 98.5%) and 1.2 log_10_ copies/mL (sensitivity, 100%; specificity, 98.5%), respectively. There was no case of false negative case in both miR-B1-5p and bkv-miR-B1-5p/miR-16. However, there was one case of false positive in miR-B1-5p and bkv-miR-B1-5p/miR-16 assays with these cut-off values.

Cut-off value for urinary BK viral load was 7.0 log_10_ copies/mL (sensitivity, 92.31%; specificity, 97.01%), which was same to currently recommended viral load cut-off [13]. However, cut-off value for plasma BK viral load was 3.5 log_10_ copies/mL (sensitivity, 84.62%; specificity, 98.51%), which was lower than that of currently recommended cut-off value (4.0 log_10_ copies/mL).

## Discussion

In our study, the prevalence of BKVN among renal allograft recipients was 2.8%, which was similar to other studies [1, 2], and most of the patients were diagnosed by indication biopsy rather than protocol biopsy. Patients included in our study were under molecular surveillance for BKVN by measuring urinary BK viral load. Nonetheless, renal biopsy was performed for most patients to differentiate BKVN from other causes of graft dysfunction, and a patient was diagnosed with BKVN without any molecular evidence of viral replication in blood or urine, revealing the limitation of viral DNA assay. Moreover, several disadvantages often reduce the usefulness of monitoring BK viral load in clinical practices, such as imperfect sensitivity in plasma DNA assay, low specificity in urinary DNA assay and variations in DNA titer among virus subtypes. These highlight the importance of novel biomarker for the diagnosis of BKVN.

Several biomarkers had been proposed as non-invasive tests for the diagnosis of BKVN in renal transplant recipients; these include heat shock protein 90-α [14], CXCL9 [15], Neutrophil gelatinase-associated lipocalin [16], urinary VP-1 [17], BK virus specific CD4 T cell [18] and urinary Haufen [19, 20]. Among these potential markers, urinary Haufen, three-dimensional cast-like polyoma virus aggregates, showed promising results as an alternative of invasive renal biopsy. Nonetheless, none of these has been introduced for clinical application in addition to currently available tests based on viral DNA.

We tested the hypothesis that exosomes derived from BKV-infected tubular cells carry viral microRNA and that the contents of exosomes shed into urine could be exploited as biomarkers of BKV replication. We confirmed for the first time that BKV microRNA can easily be monitored in urinary exosomes, suggesting a clinical application in the diagnosis of BKVN. BKV-encoded one precursor microRNA, bkv-miR-B1, generates two mature microRNA, bkv-miR-B1-3p and bkv-miR-B1-5p [4]. The bkv-miR-B1 resulted in a significant increase in the expression levels of both 3p and 5p miR-B1 in BKV-infected cells [5]. The 3p microRNA is conserved between JCV and BKV. However, the sequence of the mature 5p microRNA generated by BKV and JCV differs, and a high level of bkv-miR-B1-5p in blood is strongly associated with high levels of BK viral load and biopsy-proven BKVN [4].

The results of this study suggest that urinary exosomal 5p microRNA (bkv-miR-B1-5p) and 3p microRNA (bkv-miR-B1-3p) are significantly associated with BKVN. However, we focused on 5p microRNA as it is more specific for BKV infection [4]. The detection of high levels of bkv-miR-B1-5p in urinary exosomes was strongly associated with BKVN (Fig 2). We found a significant difference between patient groups in the levels of bkv-miR-B1-5p and bkv-miR-B1-5p/miR-16, as well as in BK viral load in plasma and urine samples. All of these parameters were significantly higher in the BKVN group. However, three cases showed false negative results with plasma BK viral load test (false negative rate of 23.1%), and urinary viral DNA test showed false positive in 3 cases (false positive rate of 4.4%). On the other hand, we demonstrated the diagnostic performance of urinary exosomal BKV microRNA was more excellent than that of plasma or urinary BK viral load; ROC analysis showed that bkv-miR-B1-5p/miR-16 had better diagnostic value compared with plasma and urinary BK viral load test (false positive rate of 1.5% and false negative rate of 0%).

In contrast to BKV microRNA, plasma and urinary BK viral load showed false negative results in patients with BKVN in our study. This is an important issue and should be addressed since all these patients showed positive SV40 staining, a histologic evidence of BK virus infection. First, the possibility that these patients were infected with the different subtypes of BK virus should be considered. Epidemiologic study found that BK virus has four subtypes (I to IV), and their geographic distribution patterns are different even within northeast Asia [21]. BK virus profiles in 68 healthy Korean were investigated in this study, revealing that subtypes I was the most commonly found BK virus (75%), followed by subtypes IV (22%), subtypes III (3%), and subtypes II (0%). Currently, our affiliated hospitals perform BKV PCR using commercial kits that can detect all subtypes of BK virus; therefore, this is not likely to be the cause of our findings. JCV nephropathy, a very rare cause of renal injury in kidney transplant recipients, could also be manifested as the same histologic features of BKVN with positive SV40 and negative BKV PCR test [22–25]. Although we did not perform molecular analysis to detect JCV, active JCV infection could be inferred from the analysis of urinary microRNAs. Given that bkv-miR-B1-3p is shared by both BKV and JCV infection while bkv-miR-B1-5p is a specific microRNA for BKV infection, patients with JCV nephropathy would demonstrate positive bkv-miR-B1-3p and negative bkv-miR-B1-5p. However, no patients in our study showed microRNA pattern that suggests JCV nephropathy. Larger epidemiologic studies as well as further research about the natural course of BKVN should be performed to elucidate these discrepancies.

Exosomes contain proteins and nucleic acids related to biosynthesis and trafficking, as well as a specific signature from the cell or tissue of origin [8]. Therefore, the contents of these vesicles have become the focus of intense research. The RNA content of exosomes is of particular diagnostic interest, as naked RNAs are not stable in the biological fluid outside vesicles due to exposure to RNase [26]. The recent discovery of microRNA inside exosomes has raised interest because microRNA has widespread impact on various fundamental and interactive cellular processes, making it promising for use as a highly specific disease biomarker [27, 28]. However, the traditional gold standard ultracentrifugation-based protocol to isolate the exosomes was not applicable for clinical samples because it is labor intensive and has highly variable outcomes. A novel spin column-based method was introduced to allow rapid and simple sample preparation and detection of low abundance transcripts in serum and plasma [29]. We were able to isolate enough exosomal RNA from 1mL of urine using this spin column-based method. The yield of the extracted exosomal RNA from l mL of urine was appropriate for clinical application, with a median value of 17.35 ng/μL urine and individual samples ranging from 7.8 to 46.2 ng/μL urine (S1 Table).

Our data show that viral microRNA levels are significantly higher in urinary exosomes from patients with BKVN. However, miR-16, a surrogate marker of bulk exosome release [12], was not significantly different between the groups. Normalization of microRNA expression by quantitative RT-PCR may be a barrier to clinical application. However, there is no consensus about the best way to normalize exosomal microRNA quantification. Pigati et al. suggested miR-16 could be a surrogate marker of exosomal contents [12], but miR-16 is also known to be an abundant microRNA in extravesicular plasma [29, 30]. In this study, because of high abundant viral microRNA in BKV cases and relatively stable expression of miR-16, normalization with miR-16 did not affect the correlation with viral DNA of plasma and urine, or the sensitivity and specificity for BKVN diagnosis.

BKV infection progresses in a stepwise manner, from urine to blood to nephropathy [31]. Therefore, appropriate and accurate diagnosis of BKV infection is important for early therapeutic intervention, including reduction of immunosuppressive treatment. Definitive diagnosis of BKVN requires detection of characteristic histological changes in the allograft biopsy [13]. However, cytopathic changes and acute tubular injuries caused by BKV typically show a focal distribution and are missed on one-third of biopsies if only a single core is evaluated [32]. The histological findings of acute rejection also share the similar feature with that of BKVN [4]. Patients with high levels of plasma BK viral load (≥4.0 log_10_ copies/mL) or urinary BK viral load (≥7.0 log_10_ copies/mL) had been generally diagnosed as presumptive BKVN [13]. However, there are various BKV DNA PCR assays, while standardized primers, probes and standards are not available.

Sensitivity and specificity were 100% and 98.5% for bkv-miR-B1-5p and 100% and 98.5% for bkv-miR-B1-5p/miR-16 using our cut-off values (5.9 and 1.2 log_10_ copies/mL, respectively). However, to determine appropriate cut-off value for diagnosis of BKVN, adequate sample size may be taken with known positive and negative cases. Another limitation of this study is that we could not determine the chronological alterations of urinary BKV microRNAs in this cross-sectional study. Finally, the differences in bkv-miR-B1-5p elevation according to the subtypes of BKV should be investigated in order to apply our results to entire kidney transplant recipients.

In conclusion, we demonstrated BK viral microRNA was highly abundant in urinary exosomal enrichment fraction in patients with BKVN, and urinary exosomal viral microRNA could be easily quantified with standard PCR method. The diagnostic power of bkv-miR-B1-5p and bkv-miR-B1-5p/miR-16 were comparable to those of plasma and urinary BK viral load. Taken together, our results suggest that urinary exosomal microRNA could be a useful biomarker for the diagnosis of BKVN. Further longitudinal prospective study is needed to confirm the discriminative power of urinary exosomal viral microRNA and to determine an appropriate cut-off value.

## Supporting information

S1 Table Extracted RNA concentration of samples. (DOCX)
