# Supplementary material for: Urinary exosomal viral microRNA as a marker of BK virus nephropathy in kidney transplant recipients
Source: PLoS One. 2017 Dec 21;12(12):e0190068. doi: 10.1371/journal.pone.0190068 (PMC5739476; doi:10.1371/journal.pone.0190068)
Supplement: S1 Table — (DOCX) [file pone.0190068.s001.docx]

**Supplementary table 1. Extracted RNA concentration of samples.**

| **Sample** | **Concentration (ng/µL)** | **A260** | **A280** |  | **260/280** | **Total RNA (µg/ µL)** |
| --- | --- | --- | --- | --- | --- | --- |
| 1 | 21.1 | 0.527 | 0.358 |  | 1.47 | 0.3165 |
| 2 | 11.1 | 0.277 | 0.161 |  | 1.72 | 0.1665 |
| 3 | 14.1 | 0.352 | 0.219 |  | 1.61 | 0.2115 |
| 4 | 13 | 0.324 | 0.209 |  | 1.55 | 0.195 |
| 5 | 17 | 0.425 | 0.276 |  | 1.54 | 0.255 |
| 6 | 22 | 0.549 | 0.382 |  | 1.44 | 0.33 |
| 7 | 9.9 | 0.249 | 0.145 |  | 1.71 | 0.1485 |
| 8 | 7.8 | 0.196 | 0.113 |  | 1.73 | 0.117 |
| 9 | 9.4 | 0.236 | 0.154 |  | 1.54 | 0.141 |
| 10 | 11 | 0.276 | 0.165 |  | 1.67 | 0.165 |
| 11 | 17.7 | 0.442 | 0.283 |  | 1.56 | 0.2655 |
| 12 | 9.9 | 0.247 | 0.163 |  | 1.51 | 0.1485 |
| 13 | 23.5 | 0.589 | 0.334 |  | 1.76 | 0.3525 |
| 14 | 27.9 | 0.697 | 0.509 |  | 1.37 | 0.4185 |
| 15 | 16.7 | 0.418 | 0.283 |  | 1.48 | 0.2505 |
| 16 | 16.5 | 0.413 | 0.274 |  | 1.51 | 0.2475 |
| 17 | 15.1 | 0.378 | 0.223 |  | 1.7 | 0.2265 |
| 18 | 23.8 | 0.596 | 0.401 |  | 1.48 | 0.357 |
| 19 | 14.7 | 0.368 | 0.229 |  | 1.61 | 0.2205 |
| 20 | 16.7 | 0.417 | 0.266 |  | 1.57 | 0.2505 |
| 21 | 13.2 | 0.329 | 0.212 |  | 1.55 | 0.198 |
| 22 | 10.9 | 0.273 | 0.175 |  | 1.56 | 0.1635 |
| 23 | 14.6 | 0.366 | 0.258 |  | 1.42 | 0.219 |
| 24 | 25.6 | 0.64 | 0.481 |  | 1.33 | 0.384 |
| 25 | 27.9 | 0.698 | 0.51 |  | 1.37 | 0.4185 |
| 26 | 15.5 | 0.387 | 0.248 |  | 1.56 | 0.2325 |
| 27 | 14.8 | 0.37 | 0.231 |  | 1.6 | 0.222 |
| 28 | 16.4 | 0.41 | 0.268 |  | 1.53 | 0.246 |
| 29 | 24.8 | 0.62 | 0.448 |  | 1.39 | 0.372 |
| 30 | 32.3 | 0.806 | 0.54 |  | 1.49 | 0.4845 |
| 31 | 23.3 | 0.583 | 0.431 |  | 1.35 | 0.3495 |
| 32 | 20.6 | 0.516 | 0.345 |  | 1.5 | 0.309 |
| 33 | 27.2 | 0.68 | 0.444 |  | 1.53 | 0.408 |
| 34 | 11.6 | 0.291 | 0.195 |  | 1.49 | 0.174 |
| 35 | 27.8 | 0.694 | 0.493 |  | 1.41 | 0.417 |
| 36 | 8 | 0.199 | 0.11 |  | 1.8 | 0.12 |
| 37 | 12 | 0.3 | 0.175 |  | 1.72 | 0.18 |
| 38 | 19.9 | 0.497 | 0.359 |  | 1.38 | 0.2985 |
| 39 | 29.4 | 0.734 | 0.491 |  | 1.49 | 0.441 |
| 40 | 12 | 0.299 | 0.178 |  | 1.68 | 0.18 |
| 41 | 10 | 0.249 | 0.158 |  | 1.58 | 0.15 |
| 42 | 42.5 | 1.064 | 0.71 |  | 1.5 | 0.6375 |
| 43 | 10.3 | 0.257 | 0.141 |  | 1.82 | 0.1545 |
| 44 | 33.1 | 0.828 | 0.546 |  | 1.52 | 0.4965 |
| 45 | 15.1 | 0.377 | 0.259 |  | 1.45 | 0.2265 |
| 46 | 14.9 | 0.373 | 0.23 |  | 1.62 | 0.2235 |
| 47 | 10.1 | 0.251 | 0.152 |  | 1.65 | 0.1515 |
| 48 | 12.7 | 0.317 | 0.186 |  | 1.7 | 0.1905 |
| 49 | 14.5 | 0.363 | 0.231 |  | 1.57 | 0.2175 |
| 50 | 16.6 | 0.415 | 0.293 |  | 1.42 | 0.249 |
| 51 | 33.7 | 0.841 | 0.529 |  | 1.59 | 0.5055 |
| 52 | 14.9 | 0.374 | 0.236 |  | 1.58 | 0.2235 |
| 53 | 16.2 | 0.406 | 0.255 |  | 1.59 | 0.243 |
| 54 | 10.8 | 0.27 | 0.163 |  | 1.66 | 0.162 |
| 55 | 10.8 | 0.269 | 0.167 |  | 1.61 | 0.162 |
| 56 | 9.3 | 0.231 | 0.137 |  | 1.69 | 0.1395 |
| 57 | 8.7 | 0.218 | 0.123 |  | 1.78 | 0.1305 |
| 58 | 25.8 | 0.645 | 0.436 |  | 1.48 | 0.387 |
| 59 | 14.5 | 0.364 | 0.226 |  | 1.61 | 0.2175 |
| 60 | 9.8 | 0.246 | 0.146 |  | 1.68 | 0.147 |
| 61 | 15.6 | 0.391 | 0.239 |  | 1.64 | 0.234 |
| 62 | 14.4 | 0.361 | 0.228 |  | 1.59 | 0.216 |
| 63 | 14.6 | 0.364 | 0.218 |  | 1.67 | 0.219 |
| 64 | 25.3 | 0.633 | 0.395 |  | 1.6 | 0.3795 |
| 65 | 22 | 0.55 | 0.355 |  | 1.55 | 0.33 |
| 66 | 13.6 | 0.339 | 0.205 |  | 1.65 | 0.204 |
| 67 | 15.3 | 0.381 | 0.245 |  | 1.56 | 0.2295 |
| 68 | 18.3 | 0.457 | 0.299 |  | 1.53 | 0.2745 |
| 69 | 12.7 | 0.317 | 0.196 |  | 1.61 | 0.1905 |
| 70 | 14.7 | 0.366 | 0.223 |  | 1.65 | 0.2205 |
| 71 | 15.8 | 0.396 | 0.246 |  | 1.61 | 0.237 |
| 72 | 10.5 | 0.263 | 0.156 |  | 1.69 | 0.1575 |
| 73 | 46.2 | 1.155 | 0.781 |  | 1.48 | 0.693 |
| 74 | 15.3 | 0.381 | 0.258 |  | 1.48 | 0.2295 |
| 75 | 16.6 | 0.416 | 0.274 |  | 1.51 | 0.249 |
| 76 | 24.8 | 0.619 | 0.477 |  | 1.3 | 0.372 |
| 77 | 11.8 | 0.295 | 0.193 |  | 1.53 | 0.177 |
| 78 | 14.6 | 0.366 | 0.244 |  | 1.5 | 0.219 |
| 79 | 12.2 | 0.305 | 0.189 |  | 1.62 | 0.183 |
| 80 | 12.8 | 0.32 | 0.197 |  | 1.63 | 0.192 |
